# Non-Fourier Heat Conduction of Nano-Films under Ultra-Fast Laser

**DOI:** 10.3390/ma16144988

**Published:** 2023-07-13

**Authors:** Yudong Mao, Shouyu Liu, Jiying Liu, Mingzhi Yu, Xinwei Li, Kaimin Yang

**Affiliations:** School of Thermal Engineering, Shandong Jianzhu University, Jinan 250101, China; maoyudong@sdjzu.edu.cn (Y.M.); 2021035121@stu.sdjzu.edu.cn (S.L.); jxl83@sdjzu.edu.cn (J.L.); ymz@sdjzu.edu.cn (M.Y.); 2022035101@stu.sdjzu.edu.cn (X.L.)

**Keywords:** non-Fourier heat conduction, ultra-fast laser, nano-films, Cattaneo–Vernotte model, dual-phase-lag model

## Abstract

The ultra-fast laser heating process of nano-films is characterized by an ultra-short duration and ultra-small space size, in which the classical Fourier law based on the hypothesis of local equilibrium is no longer applicable. Based on the Cattaneo–Vernotte (CV) model and the dual-phase-lag (DPL) model, the two-dimensional analytical solutions of heat conduction in nano-films under ultra-fast laser are obtained using the integral transformation method. The results show that there is a thermal wave phenomenon inside the film, which becomes increasingly evident as the elapse of the lag time of the temperature gradient. Moreover, the wave amplitude in the vertical direction is much larger than that in the horizontal direction of the nano-film. By comparing the numerical result of the two models, it is found that the temperature distribution inside the nano-film based on the DPL model is gentler than that of the CV model. Additionally, the temperature distribution in the two-dimensional solution is lower than that in the one-dimensional solution under the same Knudsen number. In the comparison results of the CV model, the maximum peak difference in the thermal wave reaches 75.08 K when the Knudsen number is 1.0. This demonstrates that the horizontal energy carried by the laser source significantly impacts the temperature distribution within the film.

## 1. Introduction

Micro/nano-thin films are widely used in the fields of micro-electronics, energy storage and biological medicine [1,2,3,4,5,6], among which the preparation of nano-thin films is an important technical support. The pulse duration of an ultrafast laser is from nanoseconds to femtoseconds, with the unique advantages of a short action time and a high energy density [7]. The interaction of an ultra-fast laser with a nano-thin film can solve the thermal instability during the preparation of nano-thin films and improve the preparation efficiency. Therefore, the investigation of the heat conduction in nano-thin films under the action of ultra-fast laser is of great practical significance.

The Fourier’s law, which was proposed by French scientist Fourier in 1822, established the theoretical foundation of heat conduction. It has been proved that the numerical results based on the classical Fourier law have been very appropriate to solve most heat conduction problems with low heat flux, a long thermal action time and a macro scale [8,9,10]. Chen et al. [11] investigated the repeated long-pulse laser heating of solid materials based on Fourier’s law. The research results provide a theoretical foundation and guidance for the heat conduction mechanism of repeated long-pulse lasers. Turkyilmazoglu [12] proposed a Fourier theoretical model of a laser pulse heating process for two-dimensional cylindrical materials. The surface temperature increases and attenuation inside the material is detected by irradiating the pulsed laser. The results show that the temperature will rise with the increase in the initial temperature of the material or the pulse width of the laser.

However, Fourier’s law is based on the assumption that the velocity of heat conduction is infinitely great [13]. In some special conditions, such as extremely small characteristic sizes and extremely fast process times, the speed of heat conduction is finite and Fourier’s law may no longer apply [14]. To solve the problem, the Cattaneo–Vernotte (CV) model was proposed based on the hypothesis that the speed of heat conduction is limited [15,16,17]. The model first introduced the thermal wave phenomenon and the heat flux hysteresis time *τ_q_* was introduced. Different from Fourier’s law, a hysteresis effect of temperature gradient is produced at the time *t* in a certain area of the system, where a heat flow vector appears at time *t* + *τ_q_* instead of time *t* [18]. Through this improvement, the CV model has been widely used in the fields of biology medicine [19], ultra-fast lasers [20,21] and microelectronics [22,23]. Zhang et al. [24] used the CV model to investigate the range of heat-affected under step pulse actions in a semi-infinite space. The relationship between the heat distribution and time is compared using the Fourier model and the non-Fourier model. Noroozi et al. [25] applied the CV model to investigate the heat interaction between ultra-fast lasers and thin films. They concluded that the non-linearity analysis is very significant in non-Fourier heat conduction. The observation that there is a significant difference between the Fourier and non-Fourier results emphasizes the significance of a non-Fourier model for similar problems. But the CV model only considers the heat flux density vector phase retardation, so the problem of insufficient physical basis still exists [26,27,28]. Later, Tzou [29,30] proposed the DPL model to reveal the microscopic heat conduction inside the medium from a macroscopic perspective, and to explain the hysteresis time of the heat flux vector and temperature gradient. The DPL model eliminates the assumptions in the CV model, and allows the temperature gradient ahead of the heat flux vector or the heat flux vector ahead of the temperature gradient in the process of the transient state [31]. Based on the DPL model, Majchrzak [32] calculated the internal thermal processes of cylindrical microdomains (Cr, Au) under the action of an ultra-short laser pulse and introduced the numerical model of the melting and re-solidification of the materials. Xu et al. [33] proposed an effective numerical method to solve the complexity of DPL equations. The 1-D, 2-D and 3-D models in single-phase media under laser pulse were numerically simulated. The results showed that the method was efficient in predicting the thermal behavior of laser pulse heating and other non-Fourier heat conduction problems. A.J. van der Merwe [34] used modal analysis to compare the DPL, CV and Fourier models; the results showed that the difference between the CV and the DPL is related to the values of *τ_T_* and *τ_q_*. In addition, numerical simulation methods such as the Lattice Boltzmann method (LBM), molecular dynamics (MD) and the Monte Carlo (MC) simulation have also been applied to non-Fourier heat transport problems [35,36,37,38,39]. Mao and Xu [40] investigated the one-dimensional heat conduction of ultrafast laser-heated nano-films using the LBM model and the improved CV model, respectively. The results showed that there was an obvious thermal wave phenomenon in the film, which indicates that both the analytical and numerical methods can show the thermal wave phenomenon inside the film. Although many methods are used in non-Fourier heat conduction problems, the analysis mainly focuses on 1-D. To further investigate the mechanism of non-Fourier heat conduction, the 2-D model is built using the CV and DPL models. And, the effect of relaxation time on non-Fourier heat conduction is analyzed.

This paper consists of the following parts: In Section 2, the formulas and basic models used in this paper are introduced. In Section 3, the mathematical model selected in this paper is first verified. Then, the calculation results based on the CV and DPL models are obtained, and the non-Fourier heat conduction process under different characteristic sizes of nano-film is described in the two-dimensional case. Next, the influence of hysteresis time on the heat conduction process is analyzed in the DPL model. Finally, the results of the 1-D and 2-D models are compared. In Section 4 and Section 5, the discussion and conclusion are given, respectively.

## 2. Method

### 2.1. Physical Model

**Figure 1:**
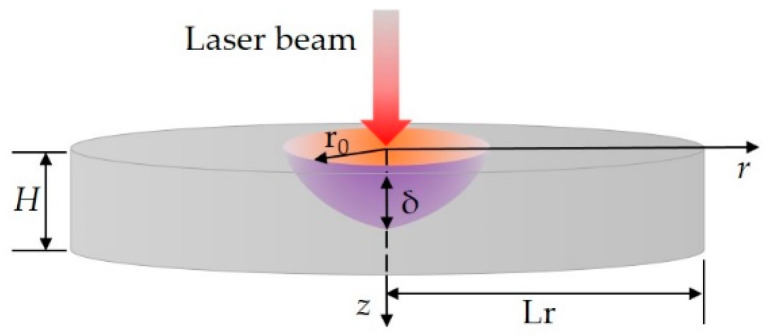
Schematic diagram of ultra-fast laser-heating nano-films.

As shown in Figure 1, the ultra-fast laser is assumed to be irradiated vertically from the center of the film. The influence radius of laser heat source and longitudinal penetration depth are r_0_ and δ, respectively. These two parameters determine the range inside the film into which the ultra-fast laser energy can be radiated. The vertical size and horizontal size of the film are *H* and L_r_, respectively. Where L_r_ = 750 nm, *H* = *l*/*Kn. Kn* is the Knudsen number, a ratio of mean free path of the energy carriers *l* to the vertical size *H*. To simplify the analysis, the film is assumed to be an adiabatic boundary.

### 2.2. Mathematical Model

The CV model and the DPL model are denoted by Equations (1) and (2), respectively [15,16,17,29,30].

(1) $$q\left(r,t+\tau_{q}\right)=-k\nabla T\left(r,t\right),$$

(2) $$q\left(r,t+\tau_{q}\right)=-k\nabla T\left(r,t+\tau_{T}\right).$$

where, *k* is the thermal conductivity of medium, ∇T is the temperature gradient which is a vector function of the time variable *t* and the position vector *r*, *τ_T_* is the phase lag time of ∇T and *τ_q_* is the phase lag time of the heat flux vector *q*.

The derivation process of the more complex DPL model is shown below. Because the derivation process of the CV model is similar to the DPL model, it is not explicitly outlined here. Combining Equation (2) with the energy conservation equation, the governing equation based on the DPL model can be expressed as Equation (3):

(3) $$\begin{array}{l} k\left(\begin{array}{l} \frac{\partial^{2}T\left(r,z,t\right)}{\partial r^{2}}+\frac{1}{r}\frac{\partial T\left(r,z,t\right)}{\partial r}+\frac{\partial^{2}T\left(r,z,t\right)}{\partial z^{2}}\\ +\tau_{T}\left(\frac{\partial^{3}T\left(r,z,t\right)}{\partial r^{2}\partial t}+\frac{1}{r}\frac{\partial^{2}T\left(r,z,t\right)}{\partial r\partial t}+\frac{\partial^{3}T\left(r,z,t\right)}{\partial z^{2}\partial t}\right) \end{array}\right)+S\left(r,z,t\right)+\tau_{q}\frac{\partial S\left(r,z,t\right)}{\partial t}\\ =C_{v}\frac{\partial T\left(r,z,t\right)}{\partial t}+C_{v}\tau_{q}\frac{\partial^{2}T\left(r,z,t\right)}{\partial t^{2}}. \end{array}$$

The definite conditions are expressed as follows:

(4) $$\frac{\partial T\left(r,z,t\right)}{\partial z}{/}_{z=0}=0,\frac{\partial T\left(r,z,t\right)}{\partial z}{/}_{z=H}=0,\frac{\partial T\left(r,z,t\right)}{\partial r}{/}_{r=L_{r}}=0,$$

(5) $$T\left(r,z,t\right){/}_{t=0}=T_{0},\frac{\partial T\left(r,z,t\right)}{\partial t}{/}_{t=0}=\frac{S\left(r,z,t\right)}{C_{v}}.$$

where *C_v_* is volumetric heat capacity and *S(r,z,t)* is the energy absorption rate of the ultra-fast laser which can be expressed as follows:

(6) $$S\left(r,z,t\right)=0.94J\frac{1-R}{t_{p}\delta }\exp\left(-\frac{z}{\delta }-\frac{r^{2}}{r_{0}^{2}}-1.992\frac{t}{t_{p}}\right).$$

where *J* is the ultra-fast laser energy density, *t_p_* is the laser pulse duration and *R* is the surface reflectivity. To solve the Equations (3)–(5), the dimensionless parameters are introduced as Equation (7):

(7) $$z^{*}=\frac{z}{H},r^{*}=\frac{r}{L_{r}},T^{*}=\frac{T-T_{0}}{T_{0}},t^{*}=\frac{t}{t_{p}},$$

Then the dimensionless governing equations are obtained:

(8) $$\begin{array}{l} a\frac{\partial^{2}T^{*}\left(r^{*},z^{*},t^{*}\right)}{\partial t^{*2}}+\frac{\partial T^{*}\left(r^{*},z^{*},t^{*}\right)}{\partial t^{*}}=\\ \frac{kt_{p}}{C_{v}}\left(\begin{array}{l} \frac{1}{L_{r}^{2}}\left(\frac{\partial^{2}T\left(r^{*},z^{*},t^{*}\right)}{\partial r^{*2}}+\frac{1}{r^{*}}\frac{\partial T\left(r^{*},z^{*},t^{*}\right)}{\partial r^{*}}\right)+\frac{1}{H^{2}}\frac{\partial^{2}T\left(r^{*},z^{*},t^{*}\right)}{\partial z^{*2}}\\ +ab\left(\frac{1}{L_{r}^{2}t_{p}}\left(\frac{\partial^{2}T\left(r^{*},z^{*},t^{*}\right)}{\partial r^{*2}}+\frac{1}{r^{*}}\frac{\partial T\left(r^{*},z^{*},t^{*}\right)}{\partial r^{*}}\right)+\frac{1}{H^{2}t_{p}}\frac{\partial^{3}T\left(r^{*},z^{*},t^{*}\right)}{\partial z^{*2}\partial t^{*}}\right) \end{array}\right)\\ +\left(1-1.992a\right)S_{0}Q^{*}\left(r^{*},z^{*},t^{*}\right), \end{array}$$

(9) $$Q^{*}\left(r^{*},z^{*},t^{*}\right)=\exp\left(-\zeta z^{*}-\varepsilon^{*2}r^{*2}-1.992t^{*}\right),$$

(10) $$S_{v}=0.94J\frac{1-R}{T_{0}C_{v}\delta },\mu =\frac{H}{\delta },\varepsilon =\frac{L_{r}}{r},a=\frac{\tau_{q}}{t_{p}},b=\frac{\tau_{T}}{\tau_{q}}.$$

where *S_v_*, μ, *ξ* and *ε* are the transition parameters with no special meaning; a is the ratio of *τ_q_* to *t_p_*, *b* is the ratio of *τ_T_* to *τ_q_*. Then, the definite solution conditions can be expressed as follows:

(11) $$\frac{\partial T^{*}\left(r^{*},z^{*},t^{*}\right)}{\partial z^{*}}{/}_{z^{*}=0}=0,\frac{\partial T^{*}\left(r^{*},z^{*},t^{*}\right)}{\partial z^{*}}{/}_{z^{*}=1}=0,\frac{\partial T^{*}\left(r^{*},z^{*},t^{*}\right)}{\partial r^{*}}{/}_{r^{*}=1}=0,$$

(12) $$T^{*}\left(r^{*},z^{*},t^{*}\right){/}_{t^{*}=0}=0;\frac{\partial T^{*}\left(r^{*},z^{*},t^{*}\right)}{\partial t^{*}}{/}_{t^{*}=0}=S_{0}Q^{*}\left(r^{*},z^{*},t^{*}\right).$$

Equations (8)–(10) are solved using the integral transformation method. In order to distinguish, one overline is added above the expression symbol ($\bar{T}$) to symbolize the integral transformation of one variable, and two overlines symbolize the integral transformation of two variables ($\bar{\bar{T^{*}}}$). The forward integral transformation and inverse integral transformation of r^*^ are shown as:

(13) $$T^{*}\left(r^{*},z^{*},t^{*}\right)=\sum_{m=0}^{\infty }\frac{J_{0}\left(\beta_{m}r^{*}\right)}{N\left(\beta_{m}\right)}\bar{T}\left(\beta_{m},z^{*},t^{*}\right),$$

(14) $$\bar{T}\left(\beta_{m},z^{*},t^{*}\right)=\int_{0}^{1}r^{*\prime }J_{0}\left(\beta_{m}r^{*}\right)T\left(r^{*},z^{*},t^{*}\right)dr^{*\prime }.$$

where $J_{0}\left(\beta_{m}r^{*}\right)$ is the Bessel functions of order zero of the first class and $\beta_{m}$ is the root of $J_{1}\left(\beta_{m}r^{*}\right)=0$, m=1,2,3…, $N\left(\beta_{m}\right)=J_{0}^{2}\left(\beta_{m}\right)/2$, $\beta_{m}\geq 0$. When m=0 and $\beta_{0}=0$, $N\left(\beta_{0}\right)=1/2$. Then the integral transformation of z^*^ are shown as follows:

(15) $$\bar{T^{*}}\left(\beta_{m},z^{*},t^{*}\right)=\sum_{n=1}^{\infty }\frac{Z\left(\eta_{n}z^{*}\right)}{N\left(\eta_{n}\right)}\bar{\bar{T^{*}}}\left(\beta_{m},\eta_{n},t^{*}\right),$$

(16) $$\bar{\bar{T^{*}}}\left(\beta_{m},\eta_{n},t^{*}\right)=\int_{0}^{1}Z^{*}\left(\eta_{n}z^{*}\right){\bar{T}}^{*}\left(\beta_{m},z^{*},t^{*}\right).$$

where n=0,1,2…, $\eta_{n}=n\pi$, $N\left(\eta_{n}\right)=\left\{\begin{array}{l} 1/2,\eta_{n}\neq 0\\ 1,\eta_{n}=0 \end{array}\right.$.

By substitution and simplification, the dimensionless governing Equations (7)–(10) can be expressed as follows:

(17) $$a\frac{\partial^{2}\bar{\bar{T^{*}}}\left(\beta_{m},\eta_{n},t^{*}\right)}{\partial t^{*2}}+\left(1+ab\gamma_{mn}\right)\frac{\partial \bar{\bar{T^{*}}}\left(\beta_{m},\eta_{n},t^{*}\right)}{\partial t^{*}}+\gamma_{mn}\bar{\bar{T^{*}}}\left(\beta_{m},\eta_{n},t^{*}\right)=\left(1-1.992a\right)S_{0}\bar{\bar{Q^{*}}}\left(\beta_{m},\eta_{n},t^{*}\right),$$

(18) $$\gamma_{mn}=\left(\frac{\beta_{m}^{2}}{L_{r}^{2}}+\frac{\eta_{n}^{2}}{H^{2}}\right)\frac{kt_{p}}{C_{v}},$$

(19) $$\bar{\bar{Q^{*}}}\left(\beta_{m},\eta_{n},t^{*}\right)=\int_{0}^{1}\begin{array}{l} r^{*}J_{0}\left(\beta_{m}r^{*}\right)\exp\left(-\varepsilon^{*2}r^{*2}\right)dr^{*}\\ \int_{0}^{1}\cos\left(\eta_{n}z^{*}\right)\exp\left(-\zeta z^{*}-1.992t^{*}\right)dz^{*}, \end{array}$$

(20) $$\bar{\bar{T^{*}}}\left(\beta_{m},\eta_{n},t^{*}\right){/}_{t^{*}=0}=0;\frac{\partial \bar{\bar{T^{*}}}\left(\beta_{m},\eta_{n},t^{*}\right)}{\partial t^{*}}{/}_{t^{*}=0}=S_{0}\bar{\bar{Q^{*}}}\left(\beta_{m},\eta_{n},t^{*}\right).$$

Equation (17) is a second-order nonhomogeneous linear ordinary differential equation; then, the expression of $\bar{\bar{T^{*}}}\left(r^{*},z^{*},t^{*}\right)$ can be obtained as:

(21) $$\bar{\bar{T^{*}}}\left(r^{*},z^{*},t^{*}\right)=\sum_{m=0}^{\infty }\sum_{n=0}^{\infty }\left[\begin{array}{l} \frac{J_{0}\left(\beta_{m}r^{*}\right)\cos\left(\eta_{n}z^{*}\right)}{N\left(\beta_{m}\right)N\left(\eta_{n}\right)}\\ \cdot \left(C_{1}F_{1}\left(\beta_{m},\eta_{n},t^{*}\right)+C_{2}F_{2}\left(\beta_{m},\eta_{n},t^{*}\right)+C\exp\left(-1.992t^{*}\right)\right) \end{array}\right].$$

Let Δ = (1 + *abγ_mn_*)^2^ − 4*aγ_mn_*, the function and parameter in the Equation (21) can be expressed as:

(22) $$C=\frac{\left(1-0.498a\right)S_{0}\int_{0}^{1}r^{*}J_{0}\left(\beta_{m}r^{*}\right)\exp\left(-\zeta z^{*}\right)dr^{*}\int_{0}^{1}\cos\left(\eta_{n}z^{*}\right)\exp\left(-\varepsilon^{*2}r^{*2}\right)dz^{*}}{\left(1.992^{2}a-1.992\left(1+ab\gamma_{mn}\right)+\gamma_{mn}\right)}.$$

(1) When Δ > 0,

(23) $$F_{1}\left(\beta_{m},\eta_{n},t^{*}\right)=e^{\frac{-\left(1+ab\gamma_{mn}\right)+\sqrt{\Delta }}{2a}t^{*}},F_{2}\left(\beta_{m},\eta_{n},t^{*}\right)=e^{\frac{-\left(1+ab\gamma_{mn}\right)-\sqrt{\Delta }}{2a}t^{*}},$$

(2) When Δ = 0,

(24) $$F_{1}\left(\beta_{m},\eta_{n},t^{*}\right)=e^{\frac{-\left(1+ab\gamma_{mn}\right)}{2a}t^{*}},F_{2}\left(\beta_{m},\eta_{n},t^{*}\right)=t^{*}e^{\frac{-\left(1+ab\gamma_{mn}\right)}{2a}t^{*}},$$

(3) When Δ < 0,

(25) $$F_{1}\left(\beta_{m},\eta_{n},t^{*}\right)=e^{\frac{-\left(1+ab\gamma_{mn}\right)}{2a}t^{*}}\cos\left(\frac{-\sqrt{\Delta }}{2a}\right),F_{2}\left(\beta_{m},\eta_{n},t^{*}\right)=e^{\frac{-\left(1+ab\gamma_{mn}\right)}{2a}t^{*}}\sin\left(\frac{-\sqrt{\Delta }}{2a}\right).$$

(26) $$\left\{\begin{array}{l} C_{1}=\frac{-C\frac{\partial F_{2}\left(\beta_{m},\eta_{n},t^{*}\right)}{\partial t^{*}}-\left(1.992C+S_{0}\int_{0}^{1}\begin{array}{l} \cos\left(\eta_{n}z^{*}\right)\exp\left(-\zeta z^{*}\right)dr^{*}\\ \int_{0}^{1}r^{*}J_{0}\left(\beta_{m}r^{*}\right)\exp\left(-\varepsilon^{*2}r^{*2}\right)dz^{*} \end{array}\right)F_{2}\left(\beta_{m},\eta_{n},t^{*}\right)}{F_{1}\left(\beta_{m},\eta_{n},t^{*}\right)\frac{\partial F_{2}\left(\beta_{m},\eta_{n},t^{*}\right)}{\partial t^{*}}-F_{2}\left(\beta_{m},\eta_{n},t^{*}\right)\frac{\partial F_{1}\left(\beta_{m},\eta_{n},t^{*}\right)}{\partial t^{*}}}\\ C_{2}=\frac{C\frac{\partial F_{1}\left(\beta_{m},\eta_{n},t^{*}\right)}{\partial t^{*}}+\left(1.992C+S_{0}\int_{0}^{1}\begin{array}{l} \cos\left(\eta_{n}z^{*}\right)\exp\left(-\zeta z^{*}\right)dr^{*}\\ \int_{0}^{1}r^{*}J_{0}\left(\beta_{m}r^{*}\right)\exp\left(-\varepsilon^{*2}r^{*2}\right)dz^{*} \end{array}\right)F_{1}\left(\beta_{m},\eta_{n},t^{*}\right)}{F_{1}\left(\beta_{m},\eta_{n},t^{*}\right)\frac{\partial F_{2}\left(\beta_{m},\eta_{n},t^{*}\right)}{\partial t^{*}}-F_{2}\left(\beta_{m},\eta_{n},t^{*}\right)\frac{\partial F_{1}\left(\beta_{m},\eta_{n},t^{*}\right)}{\partial t^{*}}} \end{array}\right..$$

Substituting Equation (21) into Equations (13) and (15), $T^{*}\left(r^{*},z^{*},t^{*}\right)$ can be obtained as follows:

(27) $$T^{*}\left(r^{*},z^{*},t^{*}\right)=\sum_{m=0}^{\infty }\sum_{n=0}^{\infty }\frac{J_{0}\left(\beta_{m}r^{*}\right)\cos\left(\eta_{n}z^{*}\right)}{N\left(\beta_{m}\right)N\left(\eta_{n}\right)}\left(C_{1}F_{1}\left(\beta_{m},\eta_{n},t^{*}\right)+C_{2}F_{2}\left(\beta_{m},\eta_{n},t^{*}\right)+C\exp\left(-1.992t^{*}\right)\right).$$

### 2.3. Cases Descriptions

Nano silicon film is selected in the present work, the physical parameters of nano-silicon thin film and an ultra-fast laser are shown in Table 1. These parameters are used in all the following calculations.

According to the above formula derivation and specific parameters, the CV and DPL models can be applied to calculate the heat conduction process inside the nano-film under the ultra-fast laser. In Table 1, the laser pulse duration *t_p_* = 0.65 ps. Combined with Equation (7), it can be seen that the laser pulse starts to launch when t* = 0, and ends when t* = 1. In order to show the heat conduction process inside the film as completely as possible, the transient temperature distributions at different dimensionless time t* are shown in the following results. In addition, Knudsen number plays an important role in terms of the scale effect, the increase in *Kn* (*Kn* = *l/H*) means the decrease in the vertical size (*H*) when the mean free path of phonons (*l*) is invariable. In the present work, the cases of *Kn* = 0.1, 0.5, 1 and 2 are mainly calculated around the vertical size of the film equivalent to the mean free path of phonons, and the corresponding vertical sizes (*H*) are 410 nm, 82 nm, 41 nm and 21 nm.

## 3. Analysis of Results

### 3.1. Authentication

To validate the methodology in the present work, a simple one-dimensional silicon film heat transfer model is established and verified with Escobar [36]. To be consistent with the mathematical model, the laser heat source is removed and the dimensionless transformation changes to the same as Chapter four in Ref [36]. Where the dimensionless temperature T* is defined as (*T* − T_2_)/(T_1_ − T_2_), *T* is the temperature at a random point in the system, T_1_ is the hot boundary temperature set at 301 K and T_2_ is the cold boundary temperature set at 300 K. Then, the same problem is solved by the DPL model in the present work.

**Figure 2:**
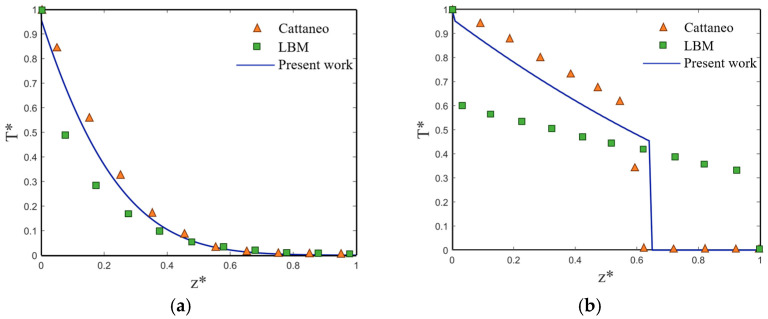
Validation of present work with different discrete data points of published numerical model [36]. (**a**) *Kn* = 0.1; (**b**) *Kn* = 1.

Figure 2a shows the transient temperature distribution at *Kn* = 0.1 in the diffusive state. All three results show that the diffusion behavior of heat transport is due to the large characteristic length and time scale of the system. The present work is well agreed with the Cattaneo and LBM, and the classical Fourier law also applies in this case [36]. When *Kn* = 1, the heat transfer changes to a transitional state as shown in Figure 2b. The results of LBM show that the temperature jumps at both ends of the film and the linear temperature distribution inside the film. The present work and Cattaneo show the phenomenon of discontinuities in the temperature distribution, which is one of the characteristics of non-Fourier heat conductions under the CV or DPL model. Compared with LBM, there are some differences in the present work. This is because the error exists in the discretization process during the implementation of the LBM numerical method, while the exact analytical solution is absolutely error-free because it is independent of the size of the domain. The above non-Fourier thermal conductivity models such as Cattaneo and DPL can be viewed as being based on the assumption that there is a time lag between heat flow and temperature gradient, so the results of the present work are more closely related to the Cattaneo. Therefore, the graph truly proves the accuracy of the current mathematical model.

### 3.2. Result of the CV Model

**Figure 3:**
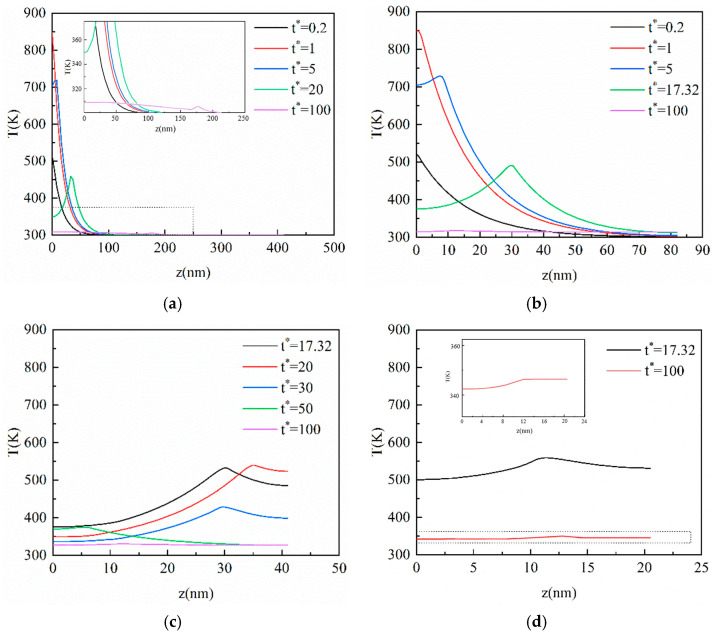
The transient temperature profile inside the film at the center of the action surface in the vertical direction based on the CV model. (**a**) *Kn* = 0.1; (**b**) *Kn* = 0.5; (**c**) *Kn* = 1; (**d**) *Kn* = 2.

In this section, the CV model is applied to analyze the heat conduction process of ultra-fast laser heating nano-thin films. As can be seen from Figure 3a, after the laser irradiates the film, the temperature of the surface subjected to the laser action rises rapidly, then decreases with the elapse of dimensionless time t*, and the heat is gradually transferred to the inside of the film. With the elapse of dimensionless time, the peak temperature point is no longer on the heated surface but appears inside the film. This phenomenon means that the heat inside the nano-thin film is transferred in the manner of a thermal wave. This is because the heat in the film cannot be diffused in time as a result of the hysteresis of the heat flux in the heating process, and the accumulation of heat can produce the phenomenon of a thermal wave. The CV model indicates that the heat transfers not in the manner of diffusion but in a limited speed, which offers a clear difference between the CV model and the Fourier model. In addition, the temperature drops rapidly after reaching the peak, such as in Figure 3a at t* = 5. This is because the energy for the temperature increase in the area near the heated surface is mainly from the laser heat source. With the depth increases in the vertical, the absorption of the laser energy decreases while the heat conduction plays a dominant role. The impact of the laser source becomes less and less as the depth is further expanded, so the heat conduction inside the film is the main mechanism controlling the temperature increase in this region.

With *Kn* increases from 0.1 to 0.5, as shown in Figure 3b, the thermal wave inside the film becomes gentler compared with *Kn* = 0.1, and the temperature inside the film is close to equilibrium at t* = 100. Figure 3c shows that the temperature peak inside the film reaches the bottom at t* = 20, and then moves in an opposite direction at t* = 30 due to the adiabatic boundary conditions. The phenomenon occurs repeatedly until the temperature inside the film reaches a stable state, although, at t* = 100 in Figure 3d, there is still a thermal wave slightly. Compared with Figure 3a–d, it can be seen that the propagation of the thermal wave inside the film becomes gentler, and the time for the temperature to approach the equilibrium state is shorter. This is because the vertical size of the film decreases as *Kn* increases and each unit length of the film absorbs more energy at the same time.

**Figure 4:**
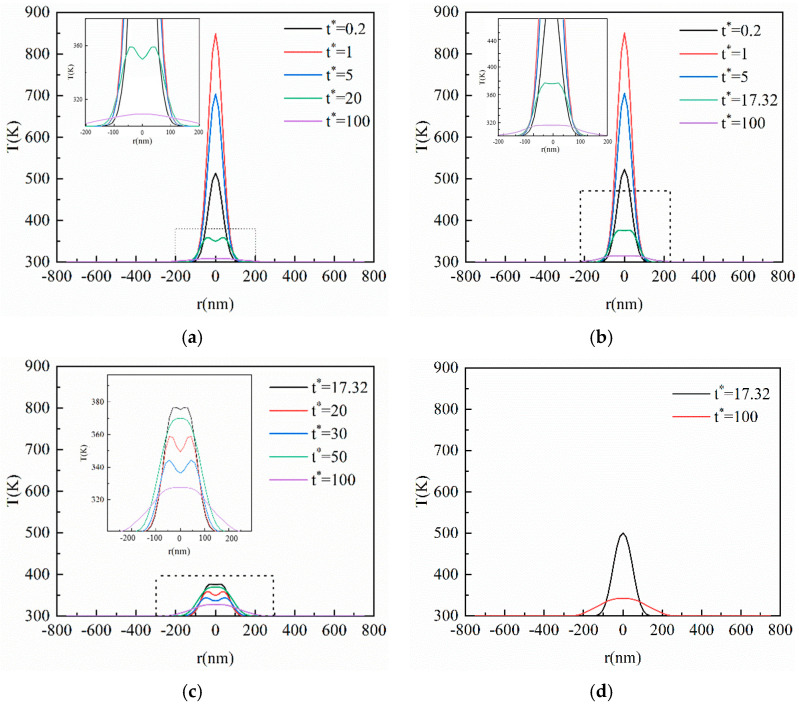
The transient temperature profile inside the film at the center of the action surface in the horizontal direction based on the CV model. (**a**) *Kn* = 0.1; (**b**) *Kn* = 0.5; (**c**) *Kn* = 1; (**d**) *Kn* = 2.

Figure 4 shows the transient temperature distribution in the horizontal direction corresponding to Figure 3. The horizontal size is 750 nm invariably, which does not change with *Kn*. As can be seen from Figure 4, the heat in the horizontal direction of the film is also transferred in the manner of a thermal wave, but the amplitude is smaller than that in the vertical. It can be seen from Equation (6) that the energy transmitted using the ultra-fast laser in the horizontal is far lower than that in the vertical direction, resulting in the heat accumulation per unit length of the film in the horizontal being much less than that in the vertical. Hence, the thermal wave amplitude in the horizontal direction is small. Additionally, when there is no thermal wave inside the film, it does not mean the heat is not transferred in the manner of a thermal wave, but the energy accumulation is not enough to make the value of the next position higher than the previous one.

From Figure 4a–d, it can be found that the horizontal size affected by the heat increases gradually with the elapse of dimensionless time, but it has not been transferred to the horizontal boundary of the film. This is because the heat transferred in the horizontal direction is much smaller than in the vertical direction. In addition, the horizontal size of the film is too large, resulting in the heat arriving at equilibrium before reaching the horizontal boundary. As is shown in Figure 3c, the transient temperature profile inside the film is transferred in an opposite direction to the vertical direction at t* = 50, which will also affect the horizontal temperature. As can be seen in Figure 4c, the phenomenon of a thermal wave in the horizontal direction disappears at t* = 50, and then the temperature at t* = 50 exceeds that at t* = 30 again. Therefore, the heat transfer in the vertical and horizontal direction will influence each other after the laser irradiates the thin film.

### 3.3. Result of the DPL Model

**Figure 5:**
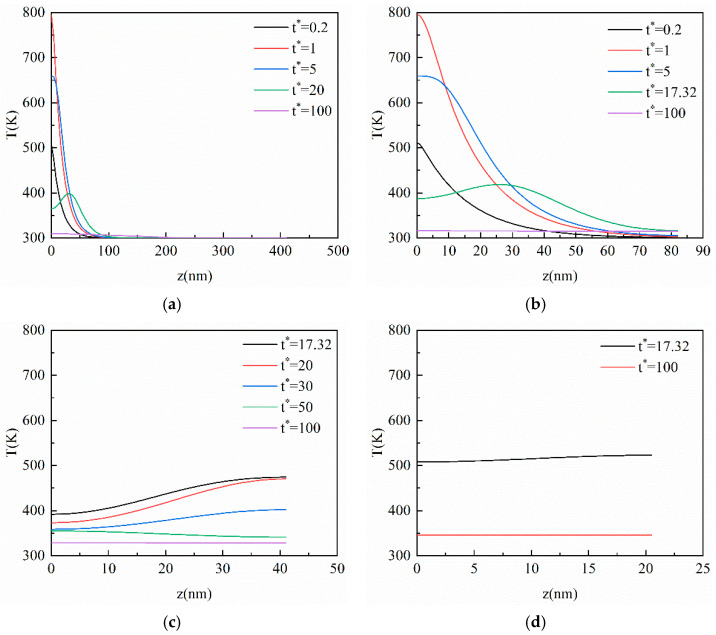
The transient temperature profile inside the film at the center of the action surface in the vertical direction based on the DPL model. (**a**) *Kn* = 0.1; (**b**) *Kn* = 0.5; (**c**) *Kn* = 1; (**d**) *Kn* = 2.

**Figure 6:**
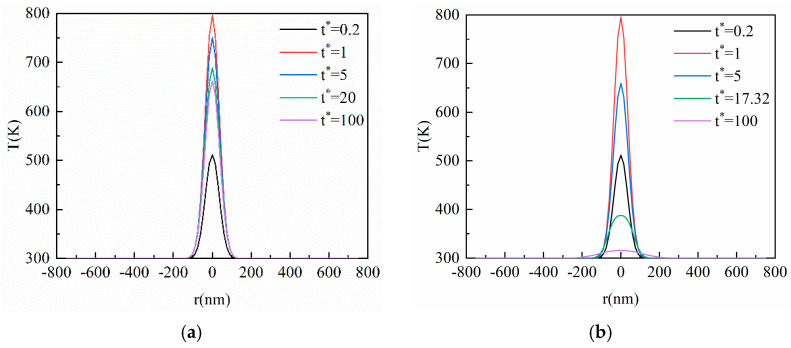

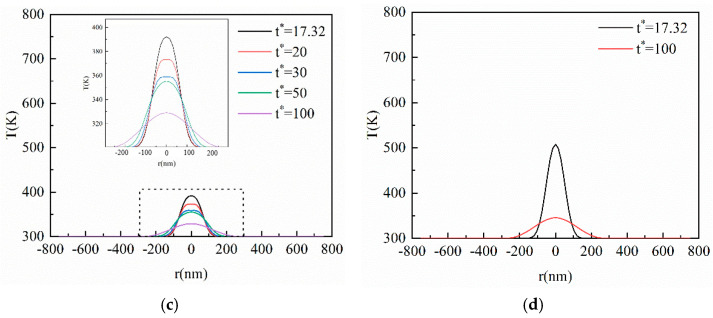
The transient temperature profile inside the film at the center of the action surface in the horizontal direction based on the DPL model. (**a**) *Kn* = 0.1; (**b**) *Kn* = 0.5; (**c**) *Kn* = 1; (**d**) *Kn* = 2.

The results using the DPL model are analyzed below. Figure 5 shows the transient temperature distribution in the vertical direction, and Figure 6 shows it in the horizontal direction corresponding to Figure 5, where *b* = 0.2 and different *Kn*s are chosen. Compared with the CV model, the temperature peak obtained based on the DPL model is relatively low under the same *Kn* and t*, the thermal wave is gentler and the propagation speed is relatively slow. That is, the peak position is slightly closer to the laser action surface of the DPL results than in that of the CV results. The reason is that the DPL model considers the hysteresis time of the heat flux and the temperature gradient between hot carriers.

Without considering the external heat source, the temperature distribution of the DPL model at dimensionless time t* is equal to the temperature distribution of the CV model at dimensionless time t* × (1 *+ b*). The detailed explanation is that the heat at the peak temperature of the film is transferred to other areas when the laser irradiates the film. Because of the influence of the hysteresis of the temperature gradient of the DPL model, the heat at peak temperature is transferred more in the film than in that of the CV model. Therefore, compared with the CV model, the temperature peak of the DPL model is lower but the temperature near the peak is higher. In other words, with the dimensionless time elapse, the thermal wave from the DPL model is relatively gentle and the position of the temperature peak changes slowly in the vertical direction.

### 3.4. Influence of the Hysteresis Time of the Temperature Gradient

**Figure 7:**
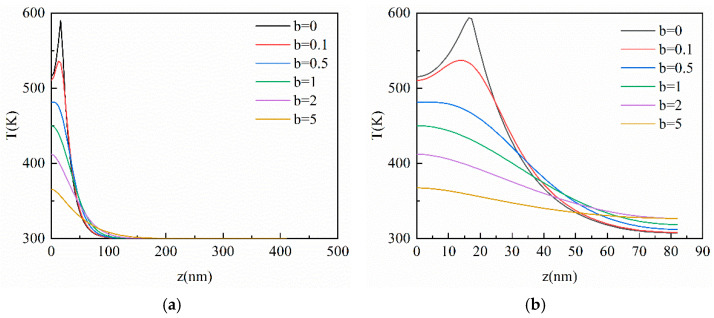
The transient temperature profile inside the film in the vertical direction at different *b* values when t* = 10. (**a**) *Kn* = 0.1; (**b**) *Kn* = 0.5.

**Figure 8:**
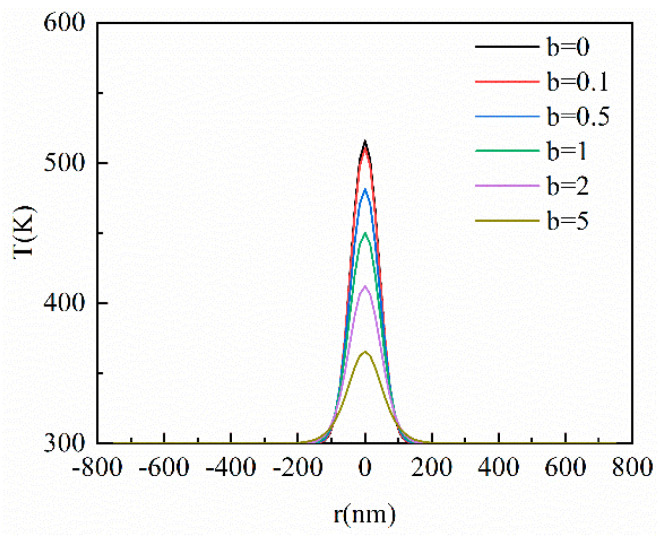
The transient temperature profile inside the film in the horizontal direction at different *b* values when t* = 10 at *Kn* = 0.1.

This section analyzes the influence of the hysteresis time of the temperature gradient on the heat conduction process by changing the *b* value, which is the ratio of *τ_T_* to *τ_q_* as shown in Equation (10). Figure 7 shows the results of different data *b* at *Kn* = 0.1 and 0.5, respectively. As is shown in Figure 7, the peak temperature becomes lower and lower with the increase in *b* value. The thermal wave phenomenon gradually disappears and the temperature distribution becomes gentler. This is because the hysteresis effect of the temperature gradient is more evident after the increase in *b* value. When *b* = 0, there is only the hysteresis of the heat flux vector inside the film, which implies that results are equivalent to the CV model. When *b* > 0, the temperature distribution will lag by *b* × t* dimensionless time because of the hysteresis time of the temperature gradient. Therefore, the temperature distribution becomes gentler following the increase in *b* value under the same t*. In addition, there is no thermal wave phenomenon in the horizontal direction as shown in Figure 8 due to the heat transferred by the ultra-fast laser source in the horizontal direction being lower than that in the vertical direction. This indicates that the appearance of the thermal wave is not only related to the hysteresis time of the temperature gradient but is greatly related to the amount of the energy transferred by the laser source.

### 3.5. Comparative between 2-D and 1-D Models

**Figure 9:**
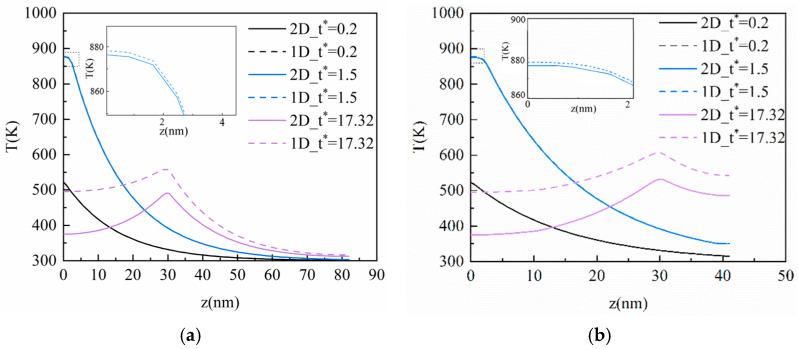
The transient temperature profile inside the film in the vertical direction of 2-D and 1-D based on the CV model. (**a**) *Kn* = 0.5; (**b**) *Kn* = 1.

**Figure 10:**
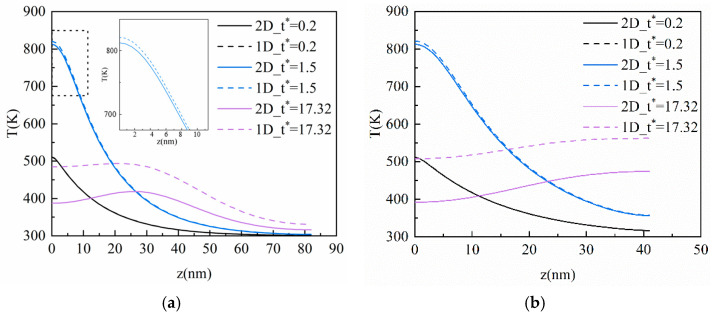
The transient temperature profile inside the film in the vertical direction of 2-D and 1-D based on the DPL model. (**a**) *Kn* = 0.5; (**b**) *Kn* = 1.

Figure 9 and Figure 10 show the comparison of temperature distribution in the vertical direction of the film between the 2-D and 1-D results of the CV model and the DPL model, respectively, where *b* = 0.2 in the DPL model. As can be seen from Figure 9, there is no evident difference between the 2-D and 1-D results at t* = 0.2 and 1.5 owing to the short heat conduction process; the ultra-fast laser source mainly acts on the vertical direction at the moment. When t* = 17.32, the difference between the two results become obvious, and the maximum difference is 120.42 K and 124.65 K in Figure 9a,b, respectively, which appears at the left boundary of the film. Although the peak of the thermal wave is reached at the same time (t* = 17.32) and position (*z* = 30 nm), the 2-D results are lower than those in the 1-D results and the peak difference of the thermal wave is 70.63 K and 75.08 K in Figure 9 (a) and (b), respectively. This is because the 2-D model takes into account the effect of the laser on the horizontal direction of the film. The conduction of heat in the horizontal direction is small at the initial time, but more and more heat in the 2-D model will transfer to the horizontal direction with the elapse of dimensionless time. In addition, as the *Kn* increases, the film contains more heat, and the difference between the 2-D and 1-D model also increases.

Figure 10 shows that the difference between the 2-D and 1-D of the DPL model is slightly greater than that of the CV model. This implies that the hysteresis time of the temperature gradient makes the influence more pronounced in the horizontal direction for the heat transfer process. By comparing the 2-D and 1-D results, it is found that the heat transfer of the horizontal dimension has extraordinary impact on the temperature distribution inside the film when the heat conduction process takes a long time. The accuracy and objectivity of the 2-D model are verified, and it is also illustrated that the horizontal heat transfer cannot be ignored in the investigation of heat transfer in thin films, nor can it be simplified into a 1-D solution.

## 4. Discussion

From the above results, we conclude that the heat is transferred in the manner of a thermal wave inside the nano-film under an ultra-fast laser based on the non-Fourier model. This conclusion is consistent with previous work on the Lattice Boltzmann method [40]. Different from the Lattice Boltzmann method, the non-Fourier model in the present work can investigate the influence of the hysteresis time of heat flux and temperature gradient on the heat transfer process. And, the heat conduction dimension is extended from 1-D to 2-D, which reveals that the heat inside the system is greatly overestimated in the 1-D model. Normally, the experiment is the most essential method to obtain the information of heat transfer inside the film, but it is almost impossible to perform a complete experiment due to its ultra-small characteristics’ size and ultra-fast physical processes. Therefore, theoretical research and simulation analysis constructed on valid mathematical models become an alternative method to exploring the mechanisms of the heat transfer process. To overcome the limitations, the next stage of our work involves conducting experiments for verification and comparison.

## 5. Conclusions

Based on the DPL model and the CV model, the non-Fourier heat conduction process of nano-films under ultra-fast laser action is investigated. Both the DPL and the CV models can show that the heat inside the nano-film is transferred in thermal waves after laser irradiation, but the temperature distribution based on the DPL model is gentler than that of the CV model.

With the decrease in the size film, the propagation of the thermal wave inside the film becomes gentler and the time for the temperature to approach the equilibrium state is shorter. And, the thermal wave in the vertical direction is more obvious than that in the horizontal direction of the film.

When the hysteresis time of the heat flux is invariable, the greater the hysteresis time of the temperature gradient and the gentler the temperature distribution. This indicates that the thermal wave phenomenon will appear in the film when the hysteresis time of the temperature gradient is less than the hysteresis time of the heat flux.

The result of the 1-D model is substantially higher than that of the 2-D model after a long period of heat conduction due to the disregard of the horizontal heat transfer. Furthermore, with the decrease in the size film, the differences will be more evident. This study verifies the accuracy and objectivity of the 2-D model and also illustrates that the horizontal heat transfer cannot be neglected in the research of heat conduction in nano-films.

## Figures and Tables

**Table 1:** Physical parameters of nano-silicon thin film and an ultrafast laser.

*T*_0_ = 300 K	*l* = 41 nm	*J* = 732 J/m^2^
*K* = 148 W/(m·K)	*τ_q_* = 6.5 ps	δ = 15.3 nm
*Cv* = 2.3 × 10^6^ J/(m^3^·K)	*tp* = 0.65 ps	*R* = 0.93

## Data Availability

Not applicable.
